# A versatile, efficient strategy for assembly of multi-fragment expression vectors in *Saccharomyces cerevisiae* using 60 bp synthetic recombination sequences

**DOI:** 10.1186/1475-2859-12-47

**Published:** 2013-05-10

**Authors:** Niels GA Kuijpers, Daniel Solis-Escalante, Lizanne Bosman, Marcel van den Broek, Jack T Pronk, Jean-Marc Daran, Pascale Daran-Lapujade

**Affiliations:** 1 Department of Biotechnology, Delft University of Technology, Julianalaan 67, Delft, 2628 BC, The Netherlands; 2 Kluyver Centre for Genomics of Industrial Fermentation, PO Box 5057, Delft, 2600 GA, The Netherlands; 3 Platform Green Synthetic Biology, Julianalaan 67, Delft, 2628 BC, The Netherlands

**Keywords:** *In vivo* assembly, *Saccharomyces cerevisiae*, Synthetic biology, Pathway engineering, Homologous recombination

## Abstract

**Background:**

*In vivo* recombination of overlapping DNA fragments for assembly of large DNA constructs in the yeast *Saccharomyces cerevisiae* holds great potential for pathway engineering on a small laboratory scale as well as for automated high-throughput strain construction. However, the current *in vivo* assembly methods are not consistent with respect to yields of correctly assembled constructs and standardization of parts required for routine laboratory implementation has not been explored. Here, we present and evaluate an optimized and robust method for *in vivo* assembly of plasmids from overlapping DNA fragments in *S. cerevisiae.*

**Results:**

To minimize occurrence of misassembled plasmids and increase the versatility of the assembly platform, two main improvements were introduced; i) the essential elements of the vector backbone (yeast episome and selection marker) were disconnected and ii) standardized 60 bp synthetic recombination sequences non-homologous with the yeast genome were introduced at each flank of the assembly fragments. These modifications led to a 100 fold decrease in false positive transformants originating from the backbone as compared to previous methods. Implementation of the 60 bp synthetic recombination sequences enabled high flexibility in the design of complex expression constructs and allowed for fast and easy construction of all assembly fragments by PCR. The functionality of the method was demonstrated by the assembly of a 21 kb plasmid out of nine overlapping fragments carrying six glycolytic genes with a correct assembly yield of 95%. The assembled plasmid was shown to be a high fidelity replica of the *in silico* design and all glycolytic genes carried by the plasmid were proven to be functional.

**Conclusion:**

The presented method delivers a substantial improvement for assembly of multi-fragment expression vectors in *S. cerevisiae*. Not only does it improve the efficiency of *in vivo* assembly, but it also offers a versatile platform for easy and rapid design and assembly of synthetic constructs. The presented method is therefore ideally suited for the construction of complex pathways and for high throughput strain construction programs for metabolic engineering purposes. In addition its robustness and ease of use facilitate the construction of any plasmid carrying two or more genes.

## Background

Restriction and ligation, complemented with the creative application of PCR, has long been the universal method for gene cloning in fundamental research and metabolic engineering [1,2]. However, the increasing size and complexity of today’s constructs in metabolic engineering has made design and construction of plasmids by these classical techniques increasingly complicated and time consuming. Several *in vitro* techniques have been developed to deal with these issues. Methods such as SLIC [3], InFusion™ [4], and Gibson’s isothermal assembly [5], enable efficient assembly of up to six overlapping DNA fragments into a plasmid. However, the efficiencies of these systems decrease at higher numbers of fragments and commercial kits are required to obtain the necessary recombinases. In contrast, *in vivo* assembly of multiple overlapping DNA fragments by homologous recombination in *Saccharomyces cerevisiae* does not exhibit these limitations [6-9]. In this method, yeast is transformed with a mixture of multiple linear DNA fragments, which assemble through homologous recombination of overlapping terminal sequences [10]. Although pioneering work in the 1980s already made use of this method to assemble circular plasmids [11], its application remained limited, probably due to the difficulties in the generation of the terminal homologous sequences required for recombination of the linear fragments. Later, *in vivo* assembly (also known as transformation associated recombination (TAR)) was used for the cloning of large DNA fragments that were resisting traditional methods based on restriction-ligation [12].

With the development of fast and cost effective chemical DNA synthesis, the method readily took off. It was shown that even single-strand 80 bp ‘stitching’ oligonucleotides overlapping the ends of adjacent fragments could be used to join DNA sequences by *in vivo* assembly in *S. cerevisiae*[13]. The full implication of these developments for TAR cloning was realized when Gibson *et al.* turned to *S. cerevisiae* to assemble four quarter genomes into a fully synthetic 583 kb *Mycoplasma genitalium* genome to overcome the size limitations of *in vitro* assembly resulting from the requirement of *E. coli* transformation [14]. This successful demonstration led to further research on *S. cerevisiae* as DNA assembly platform. It was subsequently demonstrated that a whole *M. genitalium* genome could be successfully assembled out of 25 overlapping DNA fragments in a single step [7]. In follow-up studies it was shown that as many as 38 single-stranded 200 bp oligonucleotides with 20 bp sequence overlaps could be incorporated into a linearized plasmid, thereby creating a whole new platform for gene synthesis [15]. This unparalleled efficiency of homologous recombination in *S. cerevisiae*, harnessed for high-efficiency *in vivo* assembly of linear DNA fragments, soon caught the interest of metabolic engineers [8,9]. Although TAR cloning showed many advantages, published versions of the method still yield false positive transformants at frequencies ranging from 10 to 80%, an aspect that has hitherto received comparatively little attention [16-18]. One of the main sources of incorrect assembly resides in a high incidence of transformants that contain re-circularized plasmid backbones, which contain all genetic elements required for selection and propagation [19,20]. To prevent backbone self-closure, selection procedures based on dual markers and counter-selection have been proposed [20]. Recent published protocols do not adequately deal with this incorrect assembly problem and still rely on single linearized plasmid backbones that are co-transformed to *S. cerevisiae* with a number of overlapping DNA fragments. Efficiencies measured as the percentage of clones containing the desired plasmid range from 20% to 90% and are thought to depend on the length of the homologous regions and the number of fragments to be assembled [8,9,18].

The existing methods show the potential to use *in-vivo* assembly as a standard tool for assembly of large and complex DNA constructs, but two main points should be addressed to make the system more robust and suited for large scale metabolic engineering; (i) the presence of undesired subassemblies due to regeneration of the plasmid should be reduced; and (ii) *in vivo* assembly systems should be designed in such way that replacing or swapping fragments should be feasible without extensive DNA modifications.

To meet the above requirements, the aim of the present study was to reduce the incidence of incorrect plasmid assembly and to make a robust, versatile *in vivo* assembly strategy for multi-component plasmids. To this end, the concept of a single linearized vector backbone was abandoned and replaced by separated key genetic elements involved in plasmid selection and propagation. Furthermore, specially designed 60 bp synthetic homologous recombination sequences (SHR-sequences) were implemented to enhance the versatility of the method. As a proof of principle, the method was used to assemble a 21 kb plasmid from 9 overlapping fragments, using only PCR and yeast transformation. Key factors for successful and highly efficient assembly of DNA by homologous recombination in *S. cerevisiae* are discussed*.*

## Results

### The use of single-fragment plasmid backbones results in frequent incorrect assembly

Current *in vivo* plasmid assembly methods in yeast use a linearized vector containing two elements essential for survival; i) an episome (centromere plus autonomously replicating sequence (CEN/ARS) or 2-micron origin) and ii) one or more selection marker genes. Presence of these ‘survival elements’, essential for replication and selection of the plasmid in yeast, on a single fragment, implies that re-circularization of this fragment will always generate a plasmid conferring viability to transformants in selective medium, and therefore in false positives. Circularization of such plasmid backbones can occur via two mechanisms: homologous recombination and non-homologous end joining (NHEJ) [21,22]. In the case of homologous recombination, the open end of the backbone recombines with a homologous region present in the backbone itself, which can be as short as 15 bp [23].

**Figure 1:**
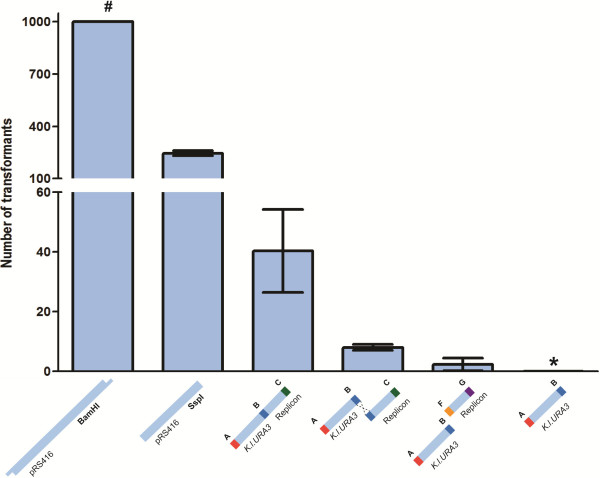
**Influence of the plasmid backbone structure on the *in vivo *assembly efficiency.** The quantification of the number of transformants obtained after transformation of 100 fmol of each of the corresponding fragment sets was based on triplicate experiments and the data presented are average ± standard deviation. (#) Transformation of the pRS416 backbone linearized by BamHI (1^st^ bar) gave so many transformants that the exact number of transformants could not be determined due to the colony density on the plates, but it exceeded 1000 transformants in all three transformations. (*) Transformation of the *K*. *lactis URA3* fragment only (rightmost bar) did not yield any transformants.

To estimate the frequency of false-positive transformants resulting from utilization of linearized plasmid backbones, we took plasmid backbones used in two recently reported *S. cerevisiae* based *in vivo* assembly methods [17,18]. Both methods made use of backbones derived from the plasmid pRS416, although in one BamHI was used to linearize pRS416, leading to a 4898 bp backbone and the other used a 2064 bp backbone resulting from the digestion of pRS416 with SspI. Yeast transformations, using the same amount of linearized plasmid DNA as described in the previous studies (100 fmol), led to over 1000 and 245 ± 14 transformants with backbone DNA restricted by BamHI and the SspI respectively (Figure 1). The higher number of clones obtained with BamHI could be explained by circularization of the backbone by cohesive-end ligation of the BamHI-cut DNA, while SspI leaves blunt ends [24]. Furthermore the BamHI fragment was 2.5 fold larger than the SspI fragment, leaving much more chance for circularization by internal recombination on short homologous sequences. These results showed that use of backbones obtained by restriction from standard yeast vectors are a serious factor in determining the fidelity of the system.

### Separation of the survival elements is important to reduce plasmid self-closure

To enhance the versatility of the *in vivo* assembly platform, we designed specific overlapping sequences (Table 1). These unique synthetic 60 bp homologous recombination sequences (SHR-sequences) were obtained by randomly combining bar-code sequences used in the *Saccharomyces* Genome Deletion Project (Table 1) [25]. The resulting 60 bp SHR-sequences can be easily attached to any DNA fragment of interest by PCR. The SHR sequences add versatility to the system, thereby creating a platform in which DNA cassettes can be easily interchanged and different combinations of genes can be effortlessly assembled.

**Table 1:** Overlapping sequences for homologous recombination

		**Comparison to *S. cerevisiae *CEN.PK113-7D genome**		
**SHR-sequence**	**Sequence 5’** ➔ **3’**	**Blast score**	**E-value**	**% GC content**
***Optimized SHR-sequences***				
A	ACTATATGTGAAGGCATGGCTATGGCACGGCAGACATTCCGCCAGATCATCAATAGGCAC	28.3	1.3	50.0
B	CACCTTTCGAGAGGACGATGCCCGTGTCTAAATGATTCGACCAGCCTAAGAATGTTCAAC	30.1	0.37	48.3
C	ACGTCTCACGGATCGTATATGCCGTAGCGACAATCTAAGAACTATGCGAGGACACGCTAG	26.5	4.5	50.0
D	ACGCATCTACGACTGTGGGTCCCGTGGAGAAATGTATGAAACCCTGTATGGAGAGTGATT	28.3	1.3	48.3
F	CATACGTTGAAACTACGGCAAAGGATTGGTCAGATCGCTTCATACAGGGAAAGTTCGGCA	28.3	1.3	46.7
G	GCCAGAGGTATAGACATAGCCAGACCTACCTAATTGGTGCATCAGGTGGTCATGGCCCTT	28.3	1.3	51.7
H	AGATTACTCTAACGCCTCAGCCATCATCGGTAATAGCTCGAATTGCTGAGAACCCGTGAC	30.1	0.37	48.3
I	TATTCACGTAGACGGATAGGTATAGCCAGACATCAGCAGCATACTTCGGGAACCGTAGGC	28.3	1.3	50.0
J	GGCCGTCATATACGCGAAGATGTCCAAGCAGGTAGAACACATAGTCTGAGCATCTCGTCG	26.5	4.5	51.7
***Endogenous sequences***				
A#	GTCGACAACCCTTAATATAACTTCGTATAATGTATGCTATACGAAGTTATTAGGTCTAGA	28.3	1.3	33.3
B#	GAGTGTTTAGAACATAATCAGTTTATCCATGGTCTATCTCTTCTTGTCGCTTTTTCTCCT	28.3	1.3	36.7
C#	TTAATTTTAAATTTTTTTGGTAGTAAAAGATGCTTATATAAGGATTTCGTATTTATTGTT	109	5e-25	18.3
D#	TAATATTTTTTCTTTTGAAAGTACTACCCACATCCGAACATTGCCACTTACATAGCGATG	109	5e-25	35.0
J#	GAACAAAGTATTTAACGCACATGTATAAATATTGTATTAAAAGGGTACCTTTATAAATAT	109	5e-25	23.3
F#	GAATAGTCTTTACACCCACAGTTTTTCGTGTGGCAGTTACTATATATTAGTAGGATATTC	109	5e-25	35.0
G#	CTAAGAAACCATTATTATCATGACATTAACCTATAAAAATAGGCGTATCACGAGGCCCTT	30.1	0.37	35.0
H#	ACGTAGGATTATTATAACTCAAAAAAATGGCATTATTCTAAGTAAGTTAAATATCCGTAA	109	5e-25	25.0
I#	TTGGCAATTTTTTGCTCTTCTATATAACAGTTGAAATTTGAATAAGAACATCTTCTCAAA	109	5e-25	26.7

Sequences were compared to the *S. cerevisiae* CEN.PK113-7D strain [26] whole genome shotgun contigs (Accession number: PRJNA52955) by BLASTN analysis [27] using BLOSUM 62 substitution matrix. The statistical significance of matches found (Blast score and E-value) are reported.

**Figure 2:**
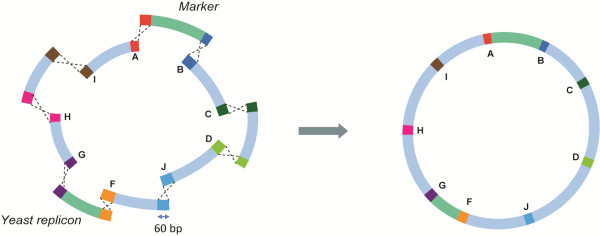
**Schematic representation of *in vivo *assembly of plasmids using 60 bp synthetic homologous recombination sequences.** The green survival fragments are essential for replication and selection.

In the present study, the classical plasmid backbone was replaced by two separate cassettes flanked by SHR sequences: one fragment containing the episome and one carrying the selection marker of the plasmid (Figure 2). Since both elements are required for a viable clone and lack any homology to each other, two independent NHEJ events are required to assemble a viable plasmid out of these fragments when co-transformed to *S. cerevisiae*. Moreover, since these fragments are flanked by SHR-sequences, interference and recombination with genomic DNA or internal regions of other assembly cassettes was expected to be less likely.

To quantify the impact of separated survival elements on the occurrence of undesired recombination events, three different cassettes were generated by PCR: (i) A *Kluyveromyces lactis URA3* cassette flanked by SHR-sequences *A* and *B* (*K.l.URA3*_*AB*_), (ii) a *CEN6/ARS4* cassette flanked by SHR-sequences *B* and *C* (*CEN6/ARS4*_*BC*_), and (iii) a *CEN6/ARS4* cassette flanked by SHR-sequences *F* and *G* (*CEN6/ARS4*_*FG*_). A fourth cassette was obtained by linking *K.l.URA3*_*AB*_ and *CEN6/ARS4*_*BC*_ by fusion PCR, leading to cassette *K.l.URA3/CEN6/ARS4*_*AC*_. Different fragment combinations containing 100 fmol of each cassette were transformed to the *S. cerevisiae* strain CEN.PK113-5D, which is auxotrophic for uracil. The fragment *K.l.URA3/CEN6/ARS4*_*AC,*_ used to mimic a linearized plasmid backbone, was also transformed alone in yeast. As expected, this fragment, which can be circularized in one single NHEJ event, resulted in a substantial number of clones (Figure 1). Still, this short fragment displayed a more than five-fold reduction in clone formation as compared to a linearized backbone (Figure 1). When the overlapping survival elements *K.l.URA3*_*AB*_ and *CEN6/ARS4*_*BC*_ were co-transformed, a 30-fold reduction was observed in the number of clones as compared to transformation with linearized backbones (Figure 1). This strong decrease can be explained by the requirement of two recombination events (if integration of the marker in chromosomal DNA is not taken into account) to generate a viable plasmid: the recombination of the separated fragments by homologous recombination and the circularization by NHEJ. When combining the non-overlapping *K.l.URA3*_*AB*_ and *CEN6/ARS4*_*FG*_ fragments hardly any clones (2±2) were obtained, which is consistent with the need for two recombination events via NHEJ to obtain a viable plasmid. Finally, transformation of only the *K.l.URA3*_*AB*_ cassette did not yield any clones, showing that integration of this cassette in the genome is extremely rare (Figure 1). These results support the hypothesis that separation of the survival elements on non-overlapping fragments reduces plasmid regeneration by at least 100-fold as compared to a linearized plasmid situation (Figure 1). Those results enabled the design of a simple, efficient *in vivo* assembly platform (Figure 2). All fragments were flanked with 60 bp SHR sequences and two survival elements, both essential for replication and selection of the plasmid, were placed opposite each other in the design.

### High efficiency and fidelity of *in vivo* assembly of a 21 kb plasmid from nine overlapping fragments

**Figure 3:**
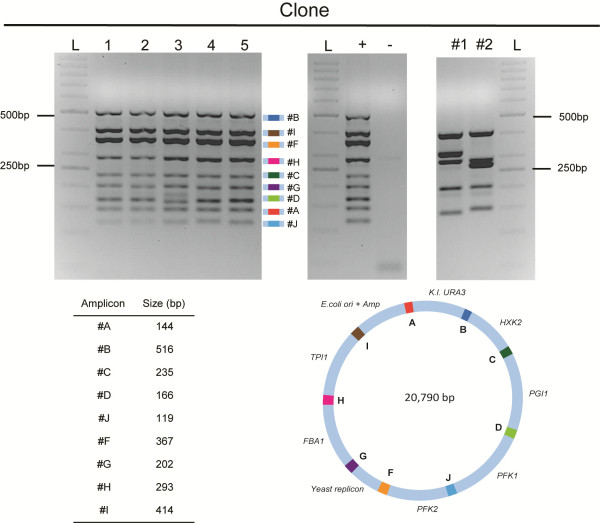
**Multiplex PCR analysis of clones obtained after co-transformation of nine overlapping fragments in *S*. *cerevisiae *and of clones obtained from control experiments.** The multiplex primer mix was designed to produce nine amplicons, ranging in size from 119–516 bp. Each amplicon covered a specific SHR-sequence. Amplicons were separated on a 2% agarose gel by electrophoresis. Lanes 1–5 represent clones obtained after transformation of a full set of fragments. As a negative control genomic DNA of CEN.PK113-5D was used (−); The later fully analyzed plasmid pUDC074 is added as a positive control (+). All nine bands were obtained in clones 1–5. The clones obtained from transformation of an incomplete mix show a completely different multiplex pattern (#1 and #2). In the lanes labeled ‘L’ a 50 bp GeneRuler ladder was loaded; sizes are indicated. In total 40 clones were analyzed and 38 multiplex patterns matched the positive control.

To test the proposed system, assembly of a 21 kb plasmid from nine DNA fragments was attempted. The fragments were amplified by PCR to add the desired SHR-sequences designed for recombination of the overlapping fragments. The nine fragments consisted of two *S. cerevisiae* survival elements, an *E. coli* amplification fragment and six expression cassettes, each containing a yeast glycolytic gene fully homologous to its genomic counterpart. The yeast survival elements *K.l.URA3*_*AB*_ and *CEN6/ARS4*_*FG*_ were constructed as described above and the *E. coli* amplification cassette *E.coli*_*IA*_ was obtained from pRS416 in the same way. The six glycolytic expression cassettes were amplified by PCR from genomic DNA of *S. cerevisiae* strain CEN.PK113-7D. Based on concentration and size measurements, ca. 100 fmol of the survival elements and 200 fmol of each of the other fragments were pooled and transformed to ca. 10^8^ yeast cells. Several controls were performed to estimate the efficiency and reliability of the technique, including: (i) transformation of the marker fragment alone, to estimate the frequency of integration of the fragment into the yeast genome, and (ii) transformation with a control mix in which a single fragment was omitted to estimate the amount of miss-assemblies. After incubation for three days on selective medium, over 1000 clones were obtained for the cells transformed with the complete set of fragments. Conversely, no clones were obtained for the cells transformed with the marker fragment only. The cells transformed with the incomplete control mix yielded six clones. High-fidelity assembly was confirmed by multiplex PCR analysis of 40 clones, randomly picked after two independent transformations. PCR with primers specifically designed to cover the SHR-sequences, produced the expected nine amplicons for 38 out of the 40 clones (Figure 3). Clones obtained by transformation with the incomplete control pool displayed aberrant multiplex PCR profiles. These results provided a strong indication for the presence of the correct assembly in 38 of the 40 tested clones, which corresponded to an extremely high efficiency of correct assembly of 95%.

To ensure that correct multiplex profiles indeed reflected the desired assembly, a single clone was randomly selected and its plasmid was isolated and named pUDC074. After amplification in *E. coli*, the sequence of pUDC074 was determined by Illumina next generation sequencing and *de novo* sequence assembly. Sequence analysis confirmed the correct assembly of the plasmid, thereby supporting the practicality of the multiplex PCR approach to screen for correct assemblies. Among the 20,790 bases of pUDC074, as few as four nucleotides were different from the original design. Of these four mutations, three single base-pair deletions were localized in the SHR-sequences. These mutations could result from erroneous homologous recombination in yeast itself, but more likely these errors were introduced prior to assembly during primer synthesis. Although HPLC purified primers were used in this study to attach the SHR-sequences, the use of PAGE purified oligonucleotides could reduce mutations resulting from synthesis. However, since the SHR-sequences are not coding, potential mutations in these overlaps are of minor concern for functionality of the assembly. The single mutation found outside the SHR-sequences was in fragment *PFK2* and caused an amino acid substitution in Pfk2 (N822K).

**Figure 4:**
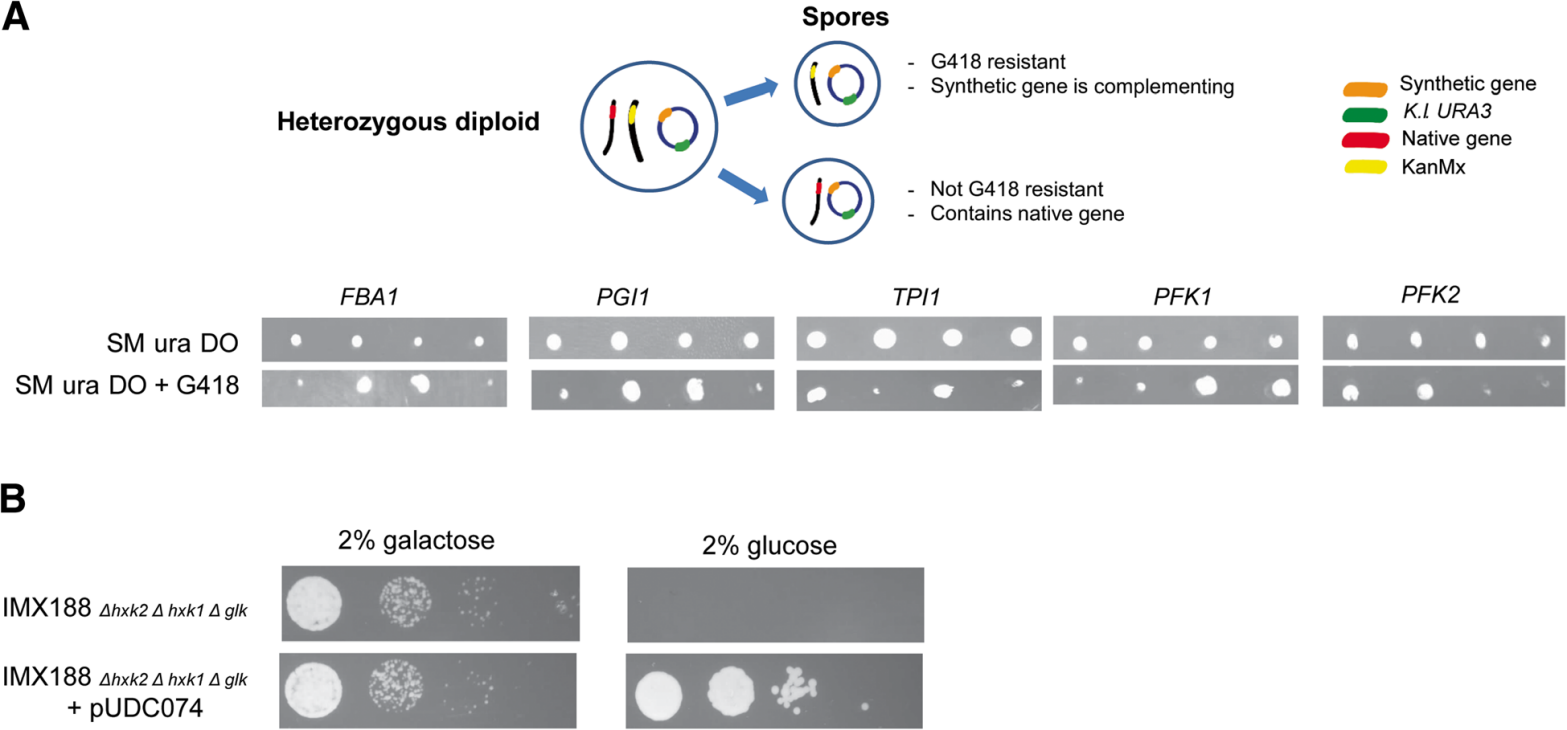
**Complementation studies of six glycolytic gene deletions with pUDC074. A)** On synthetic medium complemented with all amino acids except uracil (SM ura DO) all isolated spores from a single tetrad of a heterozygous diploid of the indicated gene can grow, proving the presence of the plasmid in all spores. Those spores were replicated to SM ura DO medium complemented with G418. Only spores containing the chromosomal deletion of the represented gene can grow due to the selection for the *KanMX* marker. Spores growing on both media confirmed the presence of a functional copy of the gene on pUDC074. **B)** Complementation study of *HXK2* with pUDC074 in a strain incapable of phosphorylating glucose. Spot plates are shown (10,000, 1000, 100, 10 cells/μl). Introduction of the plasmid restored the ability to grow on glucose as the sole carbon source.

Finally, to determine whether the presence of SHR-sequences could have an effect on the biological functionality of the proteins encoded by the six plasmid-borne glycolytic genes, a complementation study was performed (Figure 4A and B). In *S. cerevisiae*, deletion of *PGI1*[28], *TPI1*[29] or *FBA1*[30] leads to lethality and deletion of *PFK1* or *PFK2* results in severe growth impairment [31] when cultivated on glucose. The heterozygous diploids of *PGI1*, *TPI1*, *FBA1*, *PFK1* or *PFK2*[32] (Table 2) were therefore transformed with the plasmid pUDC074 and subsequently incubated in sporulation medium. After tetrad dissection, the spores containing the deletion could be selected for by the G418 resistance marker (*AgTEF2*_p_-*nptII-AgTEF2*_t_) while the presence of the plasmid was ensured by selecting for the *K.l.URA3* marker. The growth of spores in the absence of uracil and in the presence of G418 demonstrated the ability of the plasmid-borne *PGI1*, *TPI1*, *FBA1*, *PFK1* and *PFK2* genes to complement the deletion of the corresponding chromosomal gene (Figure 4A). Functionality of the assembled *HXK2* was demonstrated by restoration of growth on glucose of a glucose phosphorylation-deficient strain (IMX188, *hxk1 hxk2 glk1*; Table 2) upon transformation with pUDC074. (Figure 4B). These results demonstrated highly efficient assembly of a 21 kb plasmid out of nine fragments and that the presence of SHR-sequences has no detectable impact on the functionality of the assembled plasmid.

**Table 2:** Strains used in this study

**Strain**	**Relevant genotype**	**Source**
CEN.PK113-7D	*MATα MAL2-8c SUC2*	[26,33]
CEN.PK113-5D	*MATα ura3-52 MAL2-8c SUC2*	[33]
IMX188	*MATa ura3-52 his3-*Δ*1 leu2-3,112 MAL2-8c SUC2 glk1*Δ::*LoxP hxk1*Δ*::LoxP hxk2*Δ*::LoxP-KanMX-LoxP*	This study
IMX214	*MATa ura3-52 his3-*Δ*1 leu2-3,112 MAL2-8c SUC2 glk1*Δ*::LoxP hxk1*Δ*::LoxP hxk2*Δ*::LoxP-KanMX-LoxP + PUDC074*	This study
BY4743-Y23336	*MATa/α; ura3*Δ*0/ura3*Δ*0; pgi1*Δ*::KanMX/PGI1*	EUROSCARF^a^
BY4743-Y25893	*MATa*/*α*; *ura3*Δ*0/ura3*Δ*0;pfk1*Δ*::KanMX*/*PFK1*	EUROSCARF^a^
BY4743-Y20791	*MATa*/*α*; *ura3*Δ*0*/*ura3*Δ*0*; *pfk2*Δ::*KanMX*/*PFK2*	EUROSCARF^a^
BY4743-Y23986	*MATa*/*α*; *ura3*Δ*0*/*ura3*Δ*0*; *tpi1*Δ::*KanMX*/*TPI1*	EUROSCARF^a^
BY4743-Y24909	*MATa*/*α*; *ura3*Δ*0*/*ura3*Δ*0*; *fba1*Δ:*:KanMX*/*FBA1*	EUROSCARF^a^
Spore from Y23336 + pUDC074	*ura3*Δ*0*; *pgi1*::*kanMX pUDC074* (*K*.*I*.*URA3*(*pUG72*)*TPI HXK2 FBA1 PGI PFK1 PFK2*)	This study
Spore from Y25893 + pUDC074	*ura3*Δ*0*; *pfk1*::*kanMX pUDC074* (*K*.*I*.*URA3*(*pUG72*)*TPI HXK2 FBA1 PGI PFK1 PFK2*)	This study
Spore from Y20791 + pUDC074	*ura3*Δ*0*; *pfk2*::*kanMX pUDC074* (*K*.*I*.*URA3*(*pUG72*)*TPI HXK2 FBA1 PGI PFK1 PFK2*)	This study
Spore from Y24909 + pUDC074	*ura3*Δ*0*; *tpi1*::*kanMX pUDC074* (*K*.*I*.*URA3*(*pUG72*)*TPI HXK2 FBA1 PGI PFK1 PFK2*)	This study
Spore from Y23986 + pUDC074	*ura3*Δ*0*; *fba1*::*kanMX pUDC074* (*K*.*I*.*URA3*(*pUG72*)*TPI HXK2 FBA1 PGI PFK1 PFK2*)	This study

^a^http://web.uni-frankfurt.de/fb15/mikro/euroscarf/.

### Optimization of SHR-sequences is not critical for high efficiency of *in vivo* assembly

In published *in vivo* assembly studies, the design of the overlapping sequences across which recombination occurs does not receive much attention and often just depends on the ends of the assembled fragments. Therefore, overlaps are likely to differ in GC content and binding capacities could vary strongly between constructs. Moreover, when fragments are specifically designed for metabolic engineering in yeast, overlapping ends often share sequence identity with chromosomal sequences, since expression cassettes typically end with yeast promoter and terminator sequences. The previous experiments demonstrated that combining the separation of essential elements and the use of SHR-sequences resulted in high efficiency of *in vivo* assembly. To more precisely evaluate the contribution of the customized SHR-sequences on this high efficiency, *in vivo* assembly was also performed with fragments harboring non-optimized overlapping sequences homologous to *S. cerevisiae* genomic DNA with relatively low G/C content (18.3% to 36.7%, Table 1). Co-transformation of cassettes carrying the same glycolytic genes as in the previous experiment, but with endogenous overlapping sequences, resulted in similar colony numbers as when SHR-sequences were used. In addition, analysis of 10 randomly picked transformants by multiplex PCR did not reveal significant differences in the fidelity of *in vivo* assembly. These results demonstrated that optimization of the 60 bp overlapping sequences is not required to obtain high *in vivo* assembly efficiencies with the platform described in this study.

## Discussion

Although uncovered nearly three decades ago [11], the high efficiency of *S. cerevisiae* homologous recombination is only beginning to reveal its full potential for the assembly of large DNA constructs. *In vivo* assembly in yeast is predicted to have a large impact on laboratory practice, ranging from simple plasmid construction to engineering of complex pathways via automated high-throughput strain construction [7,8]. Despite those promising prospects, *in vivo* assembly has not yet become a standard technique in most academic laboratories. This offers unique possibilities for standardization and, simultaneously, for further optimization. While reported efficiencies of correct assembly of larger (over 15 kb) DNA constructs do not exceed 70%, efficiencies of 95% were reached in the present work for the assembly of a 21 kb construct [8,9]. Physical separation of essential elements of the plasmid backbone contributed to a strong reduction of the frequency of plasmid mis-assembly.

The high efficiency obtained with relatively short 60 bp overlaps demonstrates that, in contrast to practices and claims from recent reports [18,34], longer overlaps are not essential for efficient *in vivo* assembly. This conclusion is supported by earlier studies in which 30 to 60 bp homologous sequences were shown to lead to high recombination efficiencies in *S. cerevisiae*[32,35]. Although we anticipated that the high GC content, and therefore optimal binding properties, of the optimized SHR-sequences contributed to the high assembly efficiency obtained with the present platform, our results clearly indicate that the nature of these SHR-sequences is not an essential factor for achieving efficiencies above 90%. Therefore the increase in efficiency compared to previous studies essentially originates from the implementation of a backbone-free approach, and more specifically in the physical separation of the genetic elements on a plasmid assembly that are essential for its propagation and selection in the recipient yeast cell. Earlier studies have shown that reassembly of the backbone could make up to 95% of the obtained clones [20]. Placing the essential yeast elements on different fragments flanked by SHR-sequences and co-transforming them to *S. cerevisiae* reduced erroneous assemblies by plasmid regeneration by a factor of at least 100 (Figure 1), thereby substantially increasing the fidelity of *in vivo* assembly. Other yet unidentified factors, such as yeast strain-dependent efficiency of homologous recombination, could also have contributed to the high efficiency of assembly in the present platform and should be considered for further development of the method.

A potential downside of the proposed system was the inherent increase in the number of fragments to be transformed. However, in a standardized transformation protocol, assembly of a plasmid from 16 fragments still generated hundreds of clones (data not shown), which is more than sufficient for metabolic engineering purposes. This result indicates that increasing the number of fragments is not a serious limitation and that use of two survival elements instead of one single backbone does not have a high impact on the overall transformation efficiency.

The considerable potential of *S. cerevisiae* for pathway assembly has been recently proposed [8,9] for short pathways up to eight genes. To obtain highly productive and balanced synthetic pathways it is evident that finding the optimal combination of (heterologous) genes and expressing them at the right levels is essential [36]. Combinatorial approaches are therefore necessary and hundreds to thousands of constructs carrying different alleles expressed behind various promoters will have to be constructed in high throughput platforms. The presented approach will facilitate these strain construction programs, since assembly efficiency and robustness are bound to be key variables for high throughput strain construction, as they determine how many clones will be correctly assembled. The SHR-sequences can be designed and tested for these requirements and thus contribute to the development of these systems. Moreover, the use of SHR-sequences offers an unprecedented versatility. It is a goal of synthetic biology to create versatile platforms with libraries of interchangeable parts and pieces, as exemplified by the BioBricks concept [37]. Using the SHR sequences libraries of standardized parts, ranging from individual gene expression cassettes to fragments that carry entire pathways, can be generated and used for combinatorial assembly and subsequent screening for high-performing strains. In contrast to existing *in vivo* assembly approaches, no extensive re-designing has to be performed. Libraries of survival elements, genes and SHR-sequences will enable easy *in silico* design, straightforward *in vitro* synthesis of the fragments by PCR and efficient *in vivo* assembly.

While implementing *in vivo* assembly in our research, we have been surprised by its simplicity, ease of implementation and high efficiency. Within two years, *in vivo* assembly has almost completely replaced standard restriction/ligation protocols for construction of plasmids carrying two or more genes in our laboratory, thereby greatly accelerating strain construction and opening possibilities for strain modification that previously would have been deemed too complicated. Looking into the future, *S. cerevisiae* has the potential to be developed into an even more powerful platform. Similarly to popular *E. coli* strains, which have been extensively optimized to become extremely efficient hosts for plasmid transformation and replication, specific modifications of the yeast chassis, such as removal of the NHEJ machinery or enhancement of DNA uptake, could further extend the efficiency and fidelity of the *in vivo* assembly method.

## Conclusions

The presented method for *in vivo* assembly of multi-fragment expression vectors in *Saccharomyces cerevisiae* delivers a substantial improvement in terms of fidelity and flexibility as compared to existing methods. This improvement, achieved by replacing the plasmid backbone by standardized survival elements and by implementing the use of standardized 60 bp synthetic recombination sequences, was demonstrated by the correct assembly of a 21 kb plasmid from nine fragments with an efficiency of 95%. Ideal for the assembly of large constructs, the presented approach delivers a straightforward method for the assembly of any DNA construct carrying two or more genes and can be implemented in any molecular biology laboratory. It is our hope that the present work will contribute to standardization of *in vivo* assembly of plasmids, artificial chromosomes and synthetic genomes in *S. cerevisiae*.

## Methods

### Strains, media and DNA templates

The *S. cerevisiae* strains used in this study are listed in Table 2. Cultures for transformation were cultivated in complex media containing 10 g·l^-1^ Bacto yeast extract, 20 g·l^-1^ Bacto peptone and 20 g·l^-1^ glucose as carbon source. Synthetic medium (SM) contained per liter of demineralized water 2% agar (w/v) 5 g (NH_4_)_2_SO_4_, 3 g KH_2_PO_4_, 0.5 g MgSO_4_.7·H_2_O, and trace elements according to [38]. Filter-sterilized vitamins were added after heat sterilization of the medium at 120°C for 20 min. Glucose or galactose were separately sterilized at 110°C and added to a final concentration of 20 g·l^-1^. When required, the medium was supplemented with appropriate amounts of auxotrophic requirements [39]. Sporulation medium contained, per liter of demineralized water, 10 g potassium acetate, 1 g Bacto yeast extract and 0.5 g glucose. To rescue strain auxotrophy 10 mg leucine, 5 mg histidine, 5 mg lysine and 5 mg methionine per liter were added prior to heat sterilization. Solid medium was prepared by adding 2% agar (w/v) to the media prior to heat sterilization. Spores were grown on solid SM supplemented with Yeast Synthetic Drop-out Medium supplements without uracil (Sigma, St Louis, MO) and replica plated on the same medium supplemented with G418 (200 mg·l^-1^).

Plasmids pUG72 [40] and pRS416 [41] were maintained in *E. coli* DH5α and isolated with the GenElute™ Plasmid Miniprep Kit (Sigma). Genomic DNA was isolated from *S. cerevisiae* CEN.PK113-7D using the Qiagen 100/G kit (Qiagen, Hilden, Germany).

### Production of DNA fragments and transformation

Fragments for *in vivo* assembly were obtained from either genomic or plasmid template DNA by extension PCR using Phusion® Hot Start II High Fidelity DNA Polymerase (Thermo Fisher Scientific, Waltham, MA). Primers were HPLC purified (Sigma, St Louis, MO) and are given in Table 3. To improve the PCR efficiency, we modified the conditions recommended by the supplier, by decreasing the primer concentration from 500 nM to 200 nM and increasing the polymerase concentration from 0.02 U.μl^-1^ to 0.03 U.μl^-1^. All other conditions were chosen according to standard manufacturer instructions. The primers were designed in such a way that the annealing temperature was >65°C to minimize non-specific product formation caused by false priming. Subsequently, the amplified fragments were concentrated by chromatography using Vivacon® 500 spin columns (Sartorius Stedim, Aubagne, France). Conversely, fragments obtained from plasmid templates were submitted to an extra purification step by gel extraction to avoid contamination of the fragments by the linearized template plasmid and the ensuing formation of false positive clones. Restriction of pRS416 to obtain the linearized backbones was performed with FastDigest enzymes BamHI and SspI (Thermo Fischer Scientific) according to manufacturer instructions. The fragments *CEN6/ARS4*_*FG*_ and *E.coli*_*IA*_ were amplified from pRS416. Fragment *K.l.URA3*_*AB*_ was amplified from pUG72. Fragment *K.l.URA3/CEN6/ARS4*_*AC*_ was obtained by fusion PCR from fragments *K.l.URA3*_*AB*_ and *CEN6/ARS4*_*BC*_ using primers Fus1 and Fus2. The fragments containing the glycolytic genes were all amplified from CEN.PK113-7D genomic DNA. DNA concentrations were measured by the NanoDrop 2000 spectrophotometer (Thermo Fisher Scientific) and 200 fmol of each glycolytic gene cassette and the *E.coli*_*IA*_ fragment were pooled with 100 fmol of the *K.l.URA3*_*AB*_ and *CEN6/ARS4*_*FG*_ fragments in a final volume of 50 μl. A control pool lacking the *PFK2* cassette was created in the same way. Both pools were transformed to *S. cerevisiae* strain CEN.PK113-5D using the lithium acetate protocol [42]*.* After transformation cells were selected on synthetic medium for 3–4 days at 30°C.

**Table 3:** Primers used in this study

**Primers**	**Purification**	**Sequence 5’** ➔ **3’**
		*To add SHR*-*sequences*
E.coli Rv +A	HPLC	GTGCCTATTGATGATCTGGCGGAATGTCTGCCGTGCCATAGCCATGCCTTCACATATAGTTGCGCGGAACCCCTATTTG
E.coli Fw +I	HPLC	TATTCACGTAGACGGATAGGTATAGCCAGACATCAGCAGCATACTTCGGGAACCGTAGGCGAGAGGCGGTTTGCGTATTGG
TPI1 Rv +H	HPLC	AGATTACTCTAACGCCTCAGCCATCATCGGTAATAGCTCGAATTGCTGAGAACCCGTGACTAGTGTGAGCGGGATTTAAACTGTG
TPI1 Fw +I	HPLC	GCCTACGGTTCCCGAAGTATGCTGCTGATGTCTGGCTATACCTATCCGTCTACGTGAATAGCGAAAATGACGCTTGCAGTG
FBA1 Rv +H	HPLC	GTCACGGGTTCTCAGCAATTCGAGCTATTACCGATGATGGCTGAGGCGTTAGAGTAATCTAAAATCTCAAAAATGTGTGGGTCATTACG
FBA1 Fw +G	HPLC	GCCAGAGGTATAGACATAGCCAGACCTACCTAATTGGTGCATCAGGTGGTCATGGCCCTTAGTGCATGACAAAAGATGAGCTAGG
Cen6 Ars4 Rv +G	HPLC	AAGGGCCATGACCACCTGATGCACCAATTAGGTAGGTCTGGCTATGTCTATACCTCTGGCGACGGATCGCTTGCCTGTAAC
Cen6 Ars4 Fw +F	HPLC	CATACGTTGAAACTACGGCAAAGGATTGGTCAGATCGCTTCATACAGGGAAAGTTCGGCAGTGCCACCTGGGTCCTTTTC
Cen6 Ars4 Rv +B	HPLC	CACCTTTCGAGAGGACGATGCCCGTGTCTAAATGATTCGACCAGCCTAAGAATGTTCAACGTGCCACCTGGGTCCTTTTC
Cen6 Ars4 Fw +C	HPLC	CTAGCGTGTCCTCGCATAGTTCTTAGATTGTCGCTACGGCATATACGATCCGTGAGACGTGACGGATCGCTTGCCTGTAAC
PFK2 Rv +F	HPLC	TGCCGAACTTTCCCTGTATGAAGCGATCTGACCAATCCTTTGCCGTAGTTTCAACGTATGATAGCCATTCTCTGCTGCTTTGTTG
PFK2 Fw +J	HPLC	GGCCGTCATATACGCGAAGATGTCCAAGCAGGTAGAACACATAGTCTGAGCATCTCGTCGGAGATCCGAGGGACGTTTATTGG
PFK1 Rv +D	HPLC	ACGCATCTACGACTGTGGGTCCCGTGGAGAAATGTATGAAACCCTGTATGGAGAGTGATTTCGAGATTCCTCAATCCATACACCATTATAG
PFK1 Fw +J	HPLC	CGACGAGATGCTCAGACTATGTGTTCTACCTGCTTGGACATCTTCGCGTATATGACGGCCTGTCGTCTTCGTGAACCATTGTC
PGI1 Rv +D	HPLC	AATCACTCTCCATACAGGGTTTCATACATTTCTCCACGGGACCCACAGTCGTAGATGCGTCTGAAGAAGGCATACTACGCCAAG
PGI1 Fw +C	HPLC	ACGTCTCACGGATCGTATATGCCGTAGCGACAATCTAAGAACTATGCGAGGACACGCTAGTTCGCGACACAATAAAGTCTTCACG
HXK2 Rv +C	HPLC	CTAGCGTGTCCTCGCATAGTTCTTAGATTGTCGCTACGGCATATACGATCCGTGAGACGTGCAAGAGAAAAAAACGAGCAATTGTTAAAAG
HXK2 Fw +B	HPLC	CACCTTTCGAGAGGACGATGCCCGTGTCTAAATGATTCGACCAGCCTAAGAATGTTCAACGACGGCACCGGGAAATAAACC
URA3K.l. Rv +B	HPLC	GTTGAACATTCTTAGGCTGGTCGAATCATTTAGACACGGGCATCGTCCTCTCGAAAGGTGCTCAGAAGCTCATCGAACTGTCATC
URA3K.l. Fw + A	HPLC	ACTATATGTGAAGGCATGGCTATGGCACGGCAGACATTCCGCCAGATCATCAATAGGCACGATCCCAATACAACAGATCACGTGATC
FUS1	HPLC	ACTATATGTGAAGGCATGGCTATGG
FUS2	HPLC	CTAGCGTGTCCTCGCATAGTTC
Amp-rv + A-ctrl	HPLC	TCTAGACCTAATAACTTCGTATAGCATACATTATACGAAGTTATATTAAGGGTTGTCGACTGCGCGGAACCCCTATTTG
Amp-fw + I-ctrl	HPLC	TTGGCAATTTTTTGCTCTTCTATATAACAGTTGAAATTTGAATAAGAACATCTTCTCAAAGAGAGGCGGTTTGCGTATTGG
CEN/6ARS4-fw + F-ctrl	HPLC	GAATAGTCTTTACACCCACAGTTTTTCGTGTGGCAGTTACTATATATTAGTAGGATATTCGTGCCACCTGGGTCCTTTTC
CEN6ARS4-rv + G-ctrl	HPLC	AAGGGCCTCGTGATACGCCTATTTTTATAGGTTAATGTCATGATAATAATGGTTTCTTAGGACGGATCGCTTGCCTGTAAC
FBA1-fw + G-ctrl	HPLC	CTAAGAAACCATTATTATCATGACATTAACCTATAAAAATAGGCGTATCACGAGGCCCTTAGTGCATGACAAAAGATGAGCTAGG
FBA1-rv + H-ctrl	HPLC	TTACGGATATTTAACTTACTTAGAATAATGCCATTTTTTTGAGTTATAATAATCCTACGTAAAATCTCAAAAATGTGTGGGTCATTACG
HXK2-fw + B-ctrl	HPLC	GAGTGTTTAGAACATAATCAGTTTATCCATGGTCTATCTCTTCTTGTCGCTTTTTCTCCTGACGGCACCGGGAAATAAACC
HXK2-rv + C-ctrl	HPLC	AACAATAAATACGAAATCCTTATATAAGCATCTTTTACTACCAAAAAAATTTAAAATTAAGCAAGAGAAAAAAACGAGCAATTGTTAAAAG
K.l.URA3 -fw + A-ctrl	HPLC	GTCGACAACCCTTAATATAACTTCGTATAATGTATGCTATACGAAGTTATTAGGTCTAGAGATCCCAATACAACAGATCACGTGATC
K.l.URA3-rv + B-ctrl	HPLC	AGGAGAAAAAGCGACAAGAAGAGATAGACCATGGATAAACTGATTATGTTCTAAACACTCCTCAGAAGCTCATCGAACTGTCATC
PFK1-fw + J-ctrl	HPLC	ATATTTATAAAGGTACCCTTTTAATACAATATTTATACATGTGCGTTAAATACTTTGTTCTGTCGTCTTCGTGAACCATTGTC
PFK1-rv + D-ctrl	HPLC	TAATATTTTTTCTTTTGAAAGTACTACCCACATCCGAACATTGCCACTTACATAGCGATGTCGAGATTCCTCAATCCATACACCATTATAG
PFK2-fw + J-ctrl	HPLC	GAACAAAGTATTTAACGCACATGTATAAATATTGTATTAAAAGGGTACCTTTATAAATATGAGATCCGAGGGACGTTTATTGG
PFK2-rv + F-ctrl	HPLC	GAATATCCTACTAATATATAGTAACTGCCACACGAAAAACTGTGGGTGTAAAGACTATTCATAGCCATTCTCTGCTGCTTTGTTG
PGI-fw + C-ctrl	HPLC	TTAATTTTAAATTTTTTTGGTAGTAAAAGATGCTTATATAAGGATTTCGTATTTATTGTTTTCGCGACACAATAAAGTCTTCACG
PGI-rv + D-ctrl	HPLC	CATCGCTATGTAAGTGGCAATGTTCGGATGTGGGTAGTACTTTCAAAAGAAAAAATATTACTGAAGAAGGCATACTACGCCAAG
TPI-fw + I-ctrl	HPLC	TTTGAGAAGATGTTCTTATTCAAATTTCAACTGTTATATAGAAGAGCAAAAAATTGCCAAGCGAAAATGACGCTTGCAGTG
TPI-rv + H-ctrl	HPLC	ACGTAGGATTATTATAACTCAAAAAAATGGCATTATTCTAAGTAAGTTAAATATCCGTAATAGTGTGAGCGGGATTTAAACTGTG
		*For multipex PCR*
A Ctrl Fw	Desalted	AAATAAACAAATAGGGGTTCCGC
A Ctrl Rv	Desalted	GCAACACTCACTTCAACTTCATC
B Ctrl Fw	Desalted	TTACCACCATCCAATGCAGAC
B Ctrl Rv	Desalted	ACGGAATAGAACACGATATTTGC
C Ctrl Fw	Desalted	TCACGGGATTTATTCGTGACG
C Ctrl Rv	Desalted	GCGTCCAAGTAACTACATTATGTG
D Ctrl Fw	Desalted	ACTCGCCTCTAACCCCACG
D Ctrl Rv	Desalted	ACGGACTATAATGGTGTATGGATTG
J Ctrl Fw	Desalted	GCTTAATCTGCGTTGACAATGG
J Ctrl Rv	Desalted	CAATAAACGTCCCTCGGATCTC
F Ctrl Fw	Desalted	GACGCCATTTGGAACGAAAAAAAG
F Ctrl Rv	Desalted	ATAGCACGTGATGAAAAGGAC
G Ctrl Fw	Desalted	GCGTGTAAGTTACAGGCAAGC
G Ctrl Rv	Desalted	GCTCTTTTCTTCTGAAGGTCAATG
H Ctrl Fw	Desalted	GTTACGTGCTCAGTTGTTAGATATG
H Ctrl Rv	Desalted	GCAGAAGTGTCTGAATGTATTAAGG
I Ctrl Fw	Desalted	TGAGCCACTTAAATTTCGTGAATG
I Ctrl Rv	Desalted	GCCTTTGAGTGAGCTGATACC

### Analysis by multiplex PCR

Colonies were randomly picked and incubated overnight in appropiate medium maintaining selection pressure for the plasmid. The assemblies were isolated from 1 ml of exponentially growing culture using the GenElute™ Plasmid Miniprep Kit by adding an extra step to the supplied protocol; after harvesting the cells by centrifugation, the pellet was resuspended in 200 μl resuspension buffer supplemented with 3 μl 1000 U·ml^-1^ Zymolyase (Amsbio, Abingdon, United Kingdom) and incubated for 30 min at 37°C to digest the cell walls of the yeast cells. Further steps were performed as described by the manufacturer’s recommendations. Multiplex PCR was performed with DreamTaq PCR Master Mix (2x) (Thermo Fisher Scientific). Primers were used at a concentration of 150 nM and given in Table 3. Cycling parameters were 94°C for 3 min, then 35 cycles of 94°C for 30 s, 55°C for 90 s, and 72°C for 60 s, followed by a 10 min incubation at 72°C. Of each reaction 10 μl was loaded on a 2% agarose gel and gel electrophoresis was performed in 0.5x TBE buffer at 120 V for 40 min.

### DNA Isolation and sequencing

One positive assembly, as determined by multiplex PCR, was transformed to Electro Ten-Blue Electro-Competent cells (Agilent Technologies, Santa Clara, CA) according to the manufacturer’s instructions. From a resulting clone, plasmid DNA was isolated and analyzed by multiplex PCR. This isolated plasmid was named pUDC074. To isolate enough plasmid DNA for sequencing the plasmid was amplified in a 100 ml *E. coli* cell culture and extracted using the method of Birnboim *et al*. [43] The resulting plasmid was further purified using the Zyppy Miniprep kit (Zymoresearch, Irvine, CA). For the sequencing of pUDC074, a library of 250-bp inserts was constructed and paired-end sequenced (100 base pair reads) using an Illumina HISeq 2000 sequencer (Illumina, Eindhoven, The Netherlands) provided by Baseclear BV (Leiden, The Netherlands), generating 2 million reads. A subset of 4000 randomly picked reads, which represents a 20-fold coverage of the plasmid sequence was *de novo* assembled using IDBA [44]. IDBA was used with the following parameters: i) the paired end information was not used to scaffold the contig and ii) iterations were performed with k-mers ranged from kmin=21, kmax=99. One contig with a size of 20.8 kbase pair was assembled in which the ends were duplicated and could be merged into a circular sequence. Finally, the assembled contig was aligned to the *in silico* designed pUDC074 plasmid sequence using Clustal X in Clone Manager 9 (Sci-Ed Software**,** Cary, NC).

### Complementation studies

The required heterozygous deletion mutants (EUROSCARF, Frankfurt, Germany) were transformed with pUDC074 and clones were screened by multiplex PCR for presence of the plasmid. For each heterozygous deletion mutant a clone containing the plasmid was transferred to solid sporulation medium and incubated for 5 days. Tetrads dissection was performed as described previously [45] using a MSM 400 micromanipulator (Singer instrument, Watchet, United Kingdom). Digested asci were plated on URA drop-out medium to rescue auxotrophy and to select for spores containing the plasmid. After incubation of 3 days, colonies were replica plated on synthetic URA drop-out medium + G418 to select for presence of the KanMX marker. Plates were incubated for 2 days and checked for growth. For complementation of *HXK2*, *S. cerevisiae* strain IMX188 was transformed with pUDC074. IMX188 is deficient in glucose phoshorylation. Transformants were plated on SM with glucose as the sole carbon source and incubated for 3 days. A colony was picked and checked for presence of the plasmid by multiplex PCR. The resulting strain was named IMX214. IMX188 and IMX214 were grown overnight in SM 2% galactose. Dilutions were made for a spot plate experiment (1000, 100, 10, 1 cells / 10 μl) and 10 μl of each dilution were spotted on a SM plate with either 2% glucose (w/v) or 2% galactose (w/v) as carbon source. The plates were incubated at 30°C for 2 days and checked for growth.

## Competing interests

The authors declare that they have no competing interests.

## Author’s contributions

NGAK, DSE, JTP, JMD and PD-L designed the experimental work. NGAK and LB carried out the molecular biology experiments. MvdB performed the *de novo* sequence assembly of the plasmid. NGAK, JTP, JMD and PD-L prepared the manuscript. All authors read and approved the final manuscript.
